# Medizinische Zwillingsforschung in Deutschland

**DOI:** 10.1007/s00103-021-03400-2

**Published:** 2021-09-15

**Authors:** Paul Enck, Miriam Goebel-Stengel, Olaf Rieß, Jeannette Hübener-Schmid, Karl Oliver Kagan, Andreas Michael Nieß, Henning Tümmers, Urban Wiesing, Stephan Zipfel, Andreas Stengel, TwinHealth-Konsortium, Andreas Dufke, Sara Y. Brucker, Miriam Linneweh, Katja Fischer, Tobias Renner, Julia-Stefanie Frick, Peter Martus, Sven Nahnsen, Katja Weimer

**Affiliations:** 1 Innere Medizin VI, Psychosomatische Medizin und Psychotherapie, Universitätsklinikum Tübingen, Osianderstr. 5, 72076 Tübingen, Deutschland; 2 Klinik für Innere Medizin, Helios Klinik Rottweil, Rottweil, Deutschland; 3 Institut für Medizinische Genetik und Angewandte Genomik, Universitätsklinikum Tübingen, Tübingen, Deutschland; 4 Department für Frauengesundheit, Universitäts-Frauenklinik, Universitätsklinikum Tübingen, Tübingen, Deutschland; 5 Innere Medizin V, Sportmedizin, Universitätsklinikum Tübingen, Tübingen, Deutschland; 6 Institut für Ethik und Geschichte der Medizin, Universität Tübingen, Tübingen, Deutschland

**Keywords:** TwinHealth, Zwillingsgesundheit, Gene, Monozygot, Dizygot, Konkordanz, TwinHealth, Genes, Monozygotic, Dizygotic, Concordance

## Abstract

Nach dem Zweiten Weltkrieg wurden weltweit Zwillingskohorten aufgebaut, die inzwischen ca. 1,5 Mio. Zwillinge umfassen und zwischen 1950 und 2012 über 2748 Zwillingsstudien hervorgebracht haben. Diese Zahl steigt jedes Jahr um weitere 500 bis 1000. Die Unterrepräsentanz deutscher Zwillingsstudien in diesen Datenbanken lässt sich nicht allein durch den Missbrauch medizinischer Forschung im Nationalsozialismus erklären. Entwicklung und Ausbau großer Zwillingskohorten sind ethisch und datenschutzrechtlich eine Herausforderung. Zwillingskohorten ermöglichen jedoch die Langzeit- und Echtzeiterforschung vieler medizinischer Fragestellungen; und die Zwillingsstudien tragen auch nach der Entschlüsselung des Humangenoms erheblich zur Beantwortung der Frage nach Anlage oder Umwelt als mögliche Erkrankungsauslöser bei.

Derzeit gibt es 2 deutsche Zwillingskohorten: die biomedizinische Kohorte *HealthTwiSt* mit ca. 1500 Zwillingspaaren und *TwinLife*, eine soziologisch-psychologische Kohorte mit ca. 4000 Zwillingspaaren. Daneben gibt es krankheitsspezifische Kohorten. 2016 startete das *TwinHealth*-Konsortium der Medizinischen Fakultät der Universität Tübingen mit dem Ziel, eine forschungsoffene und nachhaltige Zwillingsforschung am Standort Tübingen zur Bearbeitung unterschiedlicher Fragestellungen zu etablieren.

Der Artikel bietet mithilfe einer systematischen Literaturrecherche und einer medizinhistorischen Betrachtung einen Überblick über die weltweite und nationale Entwicklung von Zwillingsstudien und -datenbanken der letzten 100 Jahre. Anhand der Tübinger *TwinHealth*-Initiative beleuchtet er den Aufbau eines Zwillingskollektivs und dessen juristische, ethische und Datenschutzaspekte.

## Abstract (en)

The worldwide development of twin cohorts began after World War II. These cohorts now include around 1.5 million twins, and more than 2748 twin studies have been published between 1950 and 2012. Each year, the number of twin publications increases by another 500 to 1000. The underrepresentation of German twin studies cannot be solely explained by the abuse of medical research under National Socialism. Developing and expanding large twin cohorts is a challenge in terms of both ethics and data protection. However, twin cohorts enable long-term and real-time research on many medical issues and contribute to answer the question of predisposition or environment as possible disease triggers – even after the sequencing of the human genome.

There are currently two German twin cohorts: the biomedical cohort *HealthTwiSt*, with around 1500 pairs of twins, and *TwinLife*, a sociological–psychological cohort with around 4000 pairs of twins. There are also disease-specific cohorts. The *TwinHealth* Consortium in the Faculty of Medicine at the University of Tübingen was established in 2016 with the aim of enabling open-ended and sustainable twin research in Tübingen to answer various scientific questions.

With the help of systematic literature research and medical history, this article gives an overview of the worldwide development of twin studies and databases over the last 100 years. The example of the Tübingen *TwinHealth* Initiative illuminates the structure of a twin cohort and its legal, ethical, and data protection aspects.

## Einleitung

Deutschland führt im Vergleich mit anderen Ländern, in denen es nach dem Zweiten Weltkrieg zum Aufbau großer Zwillingskohorten kam, im Bereich der Zwillingsforschung ein wissenschaftliches Schattendasein. Die skandinavischen Länder beispielsweise führen bereits Zwillingskohorten in der zweiten und dritten Generation. Von mehr als 2750 veröffentlichten Zwillingsstudien zwischen 1950 und 2012 kommen nur 40 aus Deutschland [1]; im Vergleich dazu sind im gleichen Zeitraum in den USA 945, im Vereinigten Königreich 371, in Australien 227, in den Niederlanden 190, in Dänemark 156 und in Finnland 148 Arbeiten publiziert worden.

Diese Übersicht geht den Fragen nach, warum dies so ist, welche Art von Zwillingsforschung in Deutschland betrieben wird, ob es seit der Entschlüsselung des Humangenoms im Jahr 2008 überhaupt noch einer Zwillingsforschung bedarf und welche Anforderungen an eine zeitgemäße Zwillingsforschung gestellt werden. Sie beschreibt ebenfalls die Tübinger Initiative *TwinHealth* zum Aufbau eines landesweiten Zwillingskollektivs zur Durchführung von Genexpressions‑, Genom- und Epigenomanalysen, die aber auch klassische Zwillingsstudien – und darüber hinaus vorzugsweise mit einem Zwillingsfamiliendesign – zulassen soll.

## Warum gibt es in Deutschland keine großen medizinischen Zwillingskohorten?

Im Jahr 1875 war Francis Galton, der Begründer der Eugenik, mit einer Arbeit hervorgetreten, die erstmals Zwillinge zur Bestimmung des Einflusses von Vererbung und Umwelt auf den Menschen untersuchte. Danach setzten sich Zwillingsstudien international durch. Basierend auf einem Vergleich zwischen eineiigen (monozygoten, MZ) und zweieiigen (dizygoten, DZ) Zwillingen hat Otmar Freiherr von Verschuer die „deutsche“ Tradition der Zwillingsforschung und später weltweite Standardmethode begründet. Er legte auch die ersten deutschen Zwillingsarchive an. Sein Schüler Josef Mengele verfügte als SS-Hauptsturmführer in Auschwitz über einen ungehinderten Zugang zu menschlichen Studienobjekten. Dokumentiert sind Mengeles Blutprobenentnahmen bei Minderjährigen, Untersuchungen zur Heterochromie der Augen und die Tötung verbliebener Zwillinge zum Zwecke der pathologischen Gewebeuntersuchung, nachdem ein erkrankter Zwilling verstorben war. Die Gräueltaten des Nationalsozialismus (NS) haben die Wahrnehmung und Durchführung wissenschaftlicher Experimente auf der ganzen Welt nachhaltig geprägt und die Zwillingsforschung in Deutschland nach 1945 in Verruf gebracht.

Es gibt jedoch Gründe, diese Interpretation zu hinterfragen: Mengeles Verbrechen drangen erst im letzten Drittel des 20. Jahrhunderts ins Bewusstsein einer breiteren Öffentlichkeit. Alexander Mitscherlichs berühmte Dokumentation von 1947 über den Nürnberger Ärzteprozess widmete der Zwillingsforschung kein eigenes Kapitel, im Personenverzeichnis fand Mengele keine Nennung und auch im Frankfurter Auschwitz-Prozess wurde nicht gegen Mengele verhandelt. Erst in den 1980er-Jahren entstanden Studien, die dezidiert auf Mengele fokussierten [2, 3]. Des Weiteren ist zu erklären, weshalb in der Nachkriegszeit ausgerechnet die Zwillingsforschung durch das Stigma des NS belastet wurde, während andere NS-Unrechtstaten wie Sterilisation oder Euthanasie wieder Diskursmacht erlangten. Zuletzt kollidiert die Tatsache mit dem oben aufgeführten Argument, dass just in den 1990er-Jahren, als die Erforschung der Medizin im NS Fahrt aufnahm und viele Details der Verbrechen ans Licht brachte, in Deutschland Zwillingskohorten aufgebaut wurden. Zusammengenommen existieren damit gute Gründe, sich der Genese der Zwillingsforschung im 20. Jahrhundert noch einmal aus historischer Sicht anzunehmen. Welche Konsequenzen sollten aus der Erfahrung des NS gezogen werden? Hierzu muss differenziert werden: Die Zwillingsforschung im NS war rassistisch motiviert und missachtete Menschenrechte grob. Die gegenwärtige Forschung in Deutschland verfolgt diese Ziele nicht und unterliegt zahlreichen Regulationen, die die Rechte der Beteiligten schützen. Man kann nicht sagen, dass Zwillingsforschung *an sich* stets moralisch fragwürdig ist. Wohl aber müssen die Art und die Ziele der Zwillingsforschung ethisch ausgewiesen werden. Insofern lässt sich aus der NS-Erfahrung kein Argument gegen eine ethisch vertretbare Zwillingsforschung ableiten, wohl aber eine besondere Aufmerksamkeit für Menschenrechtsverletzungen und historische Entwicklungen.

## Gegenwärtige Zwillingsforschung in Deutschland

In der zweiten Hälfte des 20. Jahrhunderts gab es in beiden Teilen Deutschlands faktisch keine Zwillingsforschung, wohl jedoch in begrenztem Umfang in Österreich [4, 5]. Erst gegen Ende des 20. Jahrhunderts kam es zum Aufbau von Zwillingskohorten, welche über viele Jahre und Generationen weitergeführt werden können, sodass Zwillinge in ihrer Lebensentwicklung, der Entstehung von Krankheiten, deren Behandlung und der Bewältigung von Krisen beobachtet werden können. Dies waren ab etwa 1996 zunächst die medizinische *HealthTwiSt*-Kohorte an der Berliner Charité (Dr. Busjahn; [6]) und einige Jahre später das Projekt *TwinLife* [7] der Universitäten Bielefeld (Profs. Riemann und Diewald) und Saarbrücken (Prof. Spinath). *TwinLife* ist ein soziologisch-psychologisches, von der Deutschen Forschungsgemeinschaft (DFG) seit 2013 gefördertes Projekt zur Untersuchung des Einflusses von Genetik und Umwelt (einschließlich Familie) auf soziale Ungleichheit. Es hat bundesweit insgesamt über 4000 Zwillingsfamilien rekrutiert und mittels Interviews und standardisierten Fragebögen soziale und psychologische Daten erfasst [7]. In einer aktuellen zweiten Phase (seit 2020) werden allerdings auch genetische Analysen eingeschlossen, die eine einmalige Entnahme von Blut- oder Speichelproben erfordern. *TwinLife* erfasst einige Gesundheitsdaten und -verhaltensweisen, aber keine speziellen Messungen zu Körperfunktionen oder zu Gesundheits- und Krankheitsstatus. Vorläufer von *TwinLife *waren lokale Zwillingsstichproben aus Jena und Bielefeld (JeTSSA, BiLSAT, GOSAT; [8–10]). Es gab außerdem eine Reihe von Begleitprojekten (CoSMoS, SOEP-Zwillingsanalysen, ChronoS) mit vergleichbaren Fragestellungen und Methoden (mit Ausnahme der Genomanalysen; [11]), die schließlich in *TwinLife* gebündelt wurden.

Die Gruppen um *HealthTwiSt* und *TwinLife* publizierten mehr als die Hälfte der genuin deutschen Zwillingsstudien, die in der im Jahr 2015 von Polderman veröffentlichten größten Metaanalyse 1950–2012 („Polderman-Datenbank“; [1]) enthalten sind. Seit 2012 sind etwa gleich viele (40) Studien aus Deutschland hinzugekommen, davon wiederum etwa die Hälfte aus diesen beiden Zwillingskohorten. Erst in den letzten 10 Jahren hat das Interesse an der Zwillingsforschung deutlich zugenommen.

### Spezielle Zwillingskollektive

Unter den Zwillingskohorten mit spezieller medizinischer Fragestellung ragt v. a. die in Hamburg und Kiel geführte Kohorte von Zwillingen mit chronisch entzündlichen Darmerkrankungen (CED), insbesondere M. Crohn [12], heraus. Die Kohorte wurde über die Patientenvereinigung Deutsche Morbus Crohn/Colitis ulcerosa Vereinigung (DCCV e. V.) rekrutiert und ging, wie viele der medizinischen Zwillingskollektive, der Frage nach, welche Gene dafür verantwortlich sind, dass auch bei MZ-Zwillingen manchmal nur ein Zwilling erkrankt (Diskordanz) und manchmal beide Zwillinge an der gleichen Erkrankung leiden (Konkordanz). Das Projekt hat eine Vielzahl von Publikationen hervorgebracht und Fortschritt in der Beantwortung der Frage gezeigt, welche Gene bei CED eine Rolle spielen. In letzter Konsequenz hat es auch gezeigt, dass die Frage *Gene oder Umwelt* selbst bei vermeintlich gesichert genetisch bedingten Erkrankungen neu diskutiert werden muss [13].

Ein weiteres Beispiel für eine Zwillingskohorte mit spezieller medizinischer Fragestellung ist die Multiple-Sklerose-Zwillingsstudie (*MS TWIN STUDY*) der Ludwig-Maximilians-Universität München und der Universität des Saarlandes. Hier konnten anhand von Mikrobiomanalysen, translationaler Forschung mit Transferexperimenten in krankheitsspezifischen Tiermodellen sowie umfassenden epigenetischen, mitochondrialen und immunologischen Untersuchungen krankheitsspezifische Erkenntnisse, aber auch für die Grundlagenforschung bei Zwillingen relevante Ergebnisse erhoben werden [14–17].

### Einzelne Zwillingsstudien

Medizinische Zwillingsstudien wurden auch in Bonn, Leipzig und Marburg/Mainz zu neurologischen Krankheitsbildern, zu metabolischen Fragestellungen bzw. zu psychiatrischen und psychosomatischen Erkrankungen, wie z. B. Essstörungen, durchgeführt [18, 19]. In diesen Fällen fällt es aber schwer, von einer Kohorte zu sprechen, da diese Gruppen offenbar nicht weiter „aufgefüllt“ werden und dadurch keine langfristige Perspektive bekommen. Stattdessen dienen sie ausschließlich zur Beantwortung einer spezifischen Fragestellung. Das trifft auch auf einzelne Zwillingsuntersuchungen bei gesunden Zwillingen zu, z. B. zur Schmerzempfindlichkeit und Placeboanalgesie in einer Tübinger Studie [20], deren Probleme bei der Rekrutierung von Zwillingen in der Bevölkerung Anlass für die Entwicklung eines breiteren Ansatzes zur Zwillingsforschung in Tübingen war.

## Gibt es nach der Entschlüsselung des Genoms noch Bedarf an Zwillingsforschung?

Zunächst einmal scheint es erstaunlich, dass es nach der Entschlüsselung des humanen Genoms im Jahr 2008 eher zu einem Anstieg als zu einer Reduktion der Zwillingsforschung gekommen ist, war doch die Zwillingsforschung in ihren Anfängen angetreten, die Frage „Anlage oder Umwelt“ eindeutig zu beantworten, gerade für komplexere soziale und psychische Merkmale wie Verhaltensweisen oder Intelligenz. Demgegenüber erschien die Frage für biologische Prozesse und Körpermerkmale eher sekundär, z. B. bei angeborenen Fehlbildungen. Aber bereits die frühen Zwillingsforscher wie von Verschuer mussten feststellen, dass die Aussage, ein Merkmal sei überwiegend vererbt, nur bedingt hilfreich ist, solange man die Regeln dieser spezifischen Vererbung (gemäß Mendel) nicht kennt [21]. Diese erfordern zusätzliche Familienuntersuchungen und die Registrierung des Auftretens des Merkmals über Generationen hinweg – darauf kann die Zwillingsforschung auch heute nicht verzichten. Nach der Entschlüsselung des Genoms kam eine weitere Erkenntnis hinzu: Eine Erkrankung, wie beispielsweise M. Crohn, wird gemäß Zwillingsuntersuchungen als überwiegend genetisch bedingt angesehen [13]; gleichzeitig zeigen Untersuchungen von diskordanten eineiigen Zwillingspaaren bis zu 200 verschiedene Genabschnitte zwischen erkranktem und nicht erkranktem Zwilling, die potenziell die Erkrankung erklären können. Es braucht daher zusätzliche Annahmen über Einflussfaktoren, die heute unter dem Oberbegriff „Epigenetik“ zusammengefasst werden: biologische Bedingungen, Lebensereignisse, soziale Umstände und individuelle Verhaltensweisen, die ein zunächst prinzipiell identisches Genom nach der Geburt noch verändern [22]. Dies zeigt, dass die Genetik die Zwillingsforschung benötigt – auch wenn beide nach wie vor allein auskommen können. Forschung zu diesen Fragestellungen könnte zwar auch in einer allgemeinen Gesundheitsstudie, wie z. B. der 2014 gestarteten Nationalen Kohorte der Helmholtz-Gemeinschaft (NAKO; https://nako.de), durchgeführt werden, aber der Anteil der Zwillingspaare (und nicht nur einzelner Zwillinge) in dieser Kohorte dürfte für die meisten Fragestellungen zu gering sein.

Dass die Genetik ein fundamentales Interesse an Zwillingen und an der Zwillingsforschung hat, ergibt sich noch aus einem anderen Umstand: Selbst bei einer seltenen, angeborenen Erkrankung wie dem Mayer-Rokitansky-Küster-Hauser-Syndrom, einer Fehlbildung der weiblichen Genitalien, finden sich unter MZ-Zwillingen einige diskordante Paare [23], was darauf hinweist, dass selbst bei MZ-Zwillingen das Genom bei der Geburt nicht zu 100 % identisch ist. MZ-Zwillinge, bei denen eine komplette Genomsequenzierung (GWAS) durchgeführt wurde, wiesen bis zu 60 unterschiedliche Mutationen auf, die teils Spontanmutationen und teils über die Eltern vererbt worden waren [24]. Möglich ist, dass 15 % der MZ-Zwillinge substanzielle Mutationen aufweisen, die nur bei einem Zwilling nachweisbar sind [25]. Anders als die klassische Zwillingsforschung konzentriert sich die Genetik, z. B. in Tübingen, dabei insbesondere auf MZ-Zwillinge mit seltenen Erkrankungen, da bei DZ-Zwillingen die genetische *A‑priori-*Variation heute noch viel zu groß ist, um eindeutige Schlussfolgerungen zuzulassen. Auf diese Weise wollen wir zeigen, dass auch die molekular- und epigenetische Forschung vom Zwillings- und Zwillingsfamiliendesign profitieren kann.

## Notwendigkeit zum Aufbau weiterer Zwillingskohorten

Eine Vielzahl systematischer und praktischer Gesichtspunkte bei der Etablierung von Zwillingskohorten ist in der Literatur, insbesondere in den Zeitschriften *Twin Research* und *Twin Research and Human Genetics* enthalten [26]. Es folgt eine Diskussion einiger dieser Aspekte aus deutscher Sicht.

Wie oben ausgeführt, braucht die Zwillingsforschung heute nicht nur ergänzend die Familienforschung, um den Erbgang über die Generationen hinweg zu erkunden, sondern auch die Genetik, um die für die Vererbung eines Merkmals verantwortlichen Gene identifizieren zu können – und sei es nur als hypothesengenerierendes Verfahren. Da aber genetische Analysen in aller Regel große Stichproben benötigen, ist schon heute in der Zwillingsforschung absehbar, dass auch große Zwillingssamples nur dann zu brauchbaren Ergebnissen kommen, wenn diese mit denen anderer Stichproben gepoolt werden [1]. Aufgrund des technischen Fortschritts bei der Genomsequenzierung entwickelt sich die Zwillingsforschung weg von der allein beschreibenden Darstellung von Unterschieden oder Gemeinsamkeiten hin zur biologischen umfassenden direkt vergleichenden Analyse von Zwillingspaaren. Die neuen Technologien erlauben potenziell auch den Einschluss multipler äußerer Faktoren (Umwelt, Belastung, Lebensführung wie Ernährung und Sport usw.) in die Ursachenforschung, indem sowohl die Aktivität der Gene (Expression) als auch die Modifikation des Genoms (Epigenom) miteinbezogen wird (sogenannte Multi-Omics-Analysen). Dies dürfte einer der Gründe sein, warum auch in jüngster Zeit immer noch neue Kohorten gegründet werden (Tab. 1). Es gibt jedoch auch Kohorten, die nicht weitergeführt werden, wie der Vergleich zwischen der Übersicht 2012 [27] und 2019 [28] ergibt.

**Table 1:** 

Land	Anzahl Kohorten	Anzahl^b^ (F = Familien)	Seit wann?	Referenz der jeweils größten Kohorte, wenn mehr als eine
*Europa*				
Belgien	2	21.200	1964	EFPTS: [30]
Dänemark	1	175.000	1950	DTR: [31]
Deutschland	2	10.000	2003	TwinLife: [7]
Vereinigtes Königreich	3	47.000	1992	TwinsUK: [32]
Finnland	2	30.500	1975	Finntwin: [33]
Israel	1	1650 (F)	2006	LIST: [34]
Italien	1	29.000	2001	ITR: [35]
Niederlande	2	255.700	1987	YNTR: [36]
Norwegen	2	42.000	2009	NTR: [37]
Portugal	1	12.300	1999	PTR: [38]
Schweden	1	216.200	1960	STR: [39]
Serbien	1	1650	2011	[40]
Spanien	1	3540	2006	MTR: [41]
Ungarn	1	2100	2007	HTR: [42]
*Amerika*				
Brasilien	1	4826	2017	Painel USP: [43]
Kanada	1	1324	1995	QNTS: [44]
Mexiko	2	145	2019	TwinsMX: [45]
USA	20	151.800	1975	MATR: [46]
*Ozeanien*				
Australien	2	90.000	1980	ATR: [47]
*Asien*				
China	3	66.000	2015	CNTR: [48]
Iran	1	1000	2017	ITR: [49]
Japan	5	39.000	1990	WJTHOMBR: [50]
Südkorea	1	4050	2001	SKTR: [51]
*Afrika*				
Guinea-Bissau	1	3600	2009	BHP: [52]
Nigeria	1	12.300	2010	NTSR: [53]
*International*				
Int. Consortia^a^	3	881.600	2013	CODATwins: [54]

^a^Enthält Zwillinge aus den nationalen Kohorten

^b^Anzahl Zwillinge, nicht Paare

Das letzte Argument ist auch einer der Gründe dafür, dass es nicht einfach ist, neue Fragestellungen (und neue Methoden) in bereits existierende Kohorten einzuarbeiten: Kohorten, die über lange Zeit aufgebaut worden sind, lassen einen Quereinstieg nur dann zu, wenn dieser für die Kohorte – und nicht (nur) für den Quereinsteiger – von Vorteil ist.

## Zwillingskohorten für die biomedizinische Forschung?

Prinzipiell sind die Erhebungsmethoden in biomedizinischen Kohorten invasiver als bei psychologisch-soziologischen Fragestellungen. Bei vielen Kohorten werden mittlerweile jedoch regelhaft Blutentnahmen durchgeführt oder Speichelproben zur DNA-Bestimmung gewonnen [28]. Höhere Belastung durch Untersuchungen reduziert die Bereitschaft zur Aufnahme in die Kohorte oder zur Teilnahme an speziellen Untersuchungen. Diese Schwelle ist bei auf *Paper-Pencil* beschränkten Methoden (Fragebögen, Tests, Interviews) deutlich niedriger. Gleichzeitig ändern sich die Untersuchungsmethoden in der biomedizinischen Forschung schneller (s. oben) und variieren zwischen den beteiligten Laboren vermutlich erheblich mehr, bevor sie zu einem medizinischen Goldstandard werden. Als Beispiel mag hier die Untersuchung des Mikrobioms im Darm dienen: Dies war vor der Jahrtausendwende vermutlich in keiner der in der Tab. 1 gelisteten Kohorten eine Fragestellung, für die Stuhlproben von Zwillingen gesammelt und gespeichert wurden. Heute aber ist dies beim gegenwärtigen Kenntnisstand zur Rolle der Darmmikrobiota für Erkrankungen ein wichtiges Forschungsthema, für das es inzwischen entsprechende Zwillingsuntersuchungen gibt [29]. Biomedizinische Kohorten müssen daher ihr Untersuchungsinstrumentarium schnell an neue Entwicklungen und Erkenntnisse anpassen, um auf dem neuesten Stand der Wissenschaft zu bleiben. Die Vielfalt der verschiedenen Fragestellungen bei den weltweiten Kohorten ist augenfällig [28].

Körperliche Untersuchungen erfordern darüber hinaus Präsenz in den Untersuchungszentren und das ist mit erhöhtem Aufwand (und Kosten) für die Zwillinge, aber auch für das Labor verbunden. Solche Untersuchungen werden daher in den meisten Kohorten auch nur für einen Teil der Kohortenmitglieder gemacht – gleichzeitig beschränkt dies die Belastung der Zwillinge, weil die Teilnahme an Untersuchungen immer auf Freiwilligkeit beruht, selbst wenn die prinzipielle Zustimmung zur Aufnahme in die Kohorte gegeben wurde (s. unten). Selektive Teilstichproben tragen aber das Risiko für einen (Selbst‑)Selektionsbias und dieser ist umso größer, je kleiner die Kohorte insgesamt ist. Die Art der Stichprobengewinnung hat weitreichende Auswirkungen auf die Zulässigkeit statistischer Methoden und das Problem der Selektivität und muss daher bei der Planung und Durchführung der Analysen Berücksichtigung finden.

Biomedizinische Zwillingskohorten haben daher in vermehrtem Umfang unvollständige Datensätze und sind somit in besonderem Maße darauf angewiesen, den biomedizinischen Kerndatensatz möglichst genau und umfänglich zu definieren und Bedingungen festzulegen, unter welchen ein Zwillingspaar überhaupt in die Kohorte aufgenommen werden kann.

## Wie wird eine Zwillingskohorte aufgebaut?

Wie oben ausgeführt, fangen Zwillingskohorten mit sehr unterschiedlichen, meist spezifischen wissenschaftlichen Fragestellungen an. Prinzipiell lassen sich jedoch 3 Wege unterscheiden:

### A: Zwillinge werden bei der Geburt registriert

Dies scheint der einfachste, aber auch der längste Weg zu einer Zwillingskohorte zu sein, es sei denn, es gibt organisatorische Unterstützung von staatlichen, regionalen oder lokalen Behörden. Diese erlauben es, eine große Zahl von Mehrlingsgeburten in einem kurzen Zeitraum zu identifizieren – wenn dies national und datenschutzrechtlich möglich ist. Deutlich im Vorteil sind hier insbesondere die skandinavischen Länder mit großen Registern, die noch dazu verbunden werden können (Geburts‑, Krankheits‑, Sozialregister), was sich in der frühen Anlage von Zwillingsregistern in diesen Ländern niederschlägt (Tab. 1). In Deutschland gibt es zwar an allen Wohnorten ein vergleichsweise gutes Melderegister, aber dort kann der Zwillingsstatus eines Individuums nur indirekt über das gleiche Geburtsdatum am gleichen Wohnort und denselben Geburtsnamen erschlossen werden. Die Kooperation der Meldebehörden ist dabei keinesfalls einheitlich und gesichert.

Naturgemäß beschränken sich Zwillingsuntersuchungen bei Neugeborenen auf biomedizinische Aspekte der Reifung und Entwicklung und pädiatrische Krankheitsbilder. Gesellschaftlich bedeutsame Erkrankungen chronischer oder degenerativer Natur treten erst viele Jahre später auf. Ein großer Vorteil von bereits während der Schwangerschaft erfassten Zwillingsföten und späterem Studieneinschluss der neugeborenen Zwillinge liegt in der möglichen Beurteilung des Schwangerschaftsverlaufs. Die Entwicklung der Kinder kann unterschiedlich und deutlich mehr durch die prä- und peripartalen Komplikationen geprägt worden sein als durch die Genetik.

Am einfachsten, weil nahezu vollständig unter lokaler Kontrolle, ist die Rekrutierung von neugeborenen Zwillingen in einer oder mehrerer Kliniken am Standort, so wie in Tübingen geplant (s. unten).

### B: Zwillinge werden mit Auftreten einer Krankheit erfasst

Dies ist vermutlich der häufigste Weg zu einer biomedizinischen Zwillingskohorte, was seine Ursache in der großen Bandbreite medizinischer Fachrichtungen hat. Die Situation ist deutlich einheitlicher in der Psychologie, wo es immer (noch) um die Frage der Anlage oder Umwelt (*Nature versus Nurture*) psychischer Dispositionen jenseits von psychischen Erkrankungen geht.

Zentral bei allen Erkrankungen von Zwillingen ist dabei die Frage der Konkordanz oder Diskordanz, wobei Diskordanz insgesamt häufiger ist und insofern eine Besonderheit darstellt, weil damit die Frage verbunden ist, ob der verbleibende Zwilling eine erhöhte Wahrscheinlichkeit hat, zukünftig zu erkranken. Bei schwerwiegenden, lebensbedrohlichen Krankheiten stellt dies gerade bei MZ-Zwillingen auch eine erhebliche psychische Belastung dar.

Hinzu kommt, dass Konkordanz in den meisten Fällen nur ein relatives Maß ist, wie Tab. 2 zu klassischen neurologischen Erkrankungen zeigt: Sie kann in Abhängigkeit von der Erkrankung zwischen 3 % und 30 % variieren, d. h., selbst wenn sie bei MZ- höher ist als bei DZ-Zwillingen (und damit auf eine genetische Abhängigkeit deutet), treten mehrheitlich (in 70–97 % der Fälle) die Krankheiten nur bei einem Zwilling auf.

**Table 2:** 

Krankheit	Anzahl Studien	Anzahl Zwillingspaare^a^ (MZ und DZ)	Konkordanz MZ – DZ Prozent	Genetik vs. Umwelt Prozent	Literatur
M. Parkinson	4	607	11 – 4	40 – 60	[55]
M. Alzheimer	9	392	(73 – 45)	60–80/20–40	[56]
Migräne	14	211	33 – 12	79 – 21	[57]
Apoplex	6	906	3,1 – 0,3	40 – 60	[58]
Epilepsie	9	1323	18 – 5	(80)	[59]
MS	4	171	24 – 3	(15 – 75)	[60]

Zahlen in Klammern zeigen an, dass sie aus anderen Studien stammen

*MZ* monozygote Zwillinge, *DZ* dizygote Zwillinge, *MS* multiple Sklerose

^a^In der Regel sind ca. 2 Drittel der Paare dizygot

Der Tübinger Weg wird hier ein Sonderweg sein, der unseres Wissens bislang noch nicht beschritten wurde (s. unten).

### C: Zwillinge werden in der Bevölkerung rekrutiert

Dies ist ein häufiger Weg, um gesunde Zwillinge zu rekrutieren – sei es als Versuchspersonen für physiologische/medizinische Untersuchungen oder als Kontrollgruppe für kranke Zwillinge, die über den unter B genannten Weg rekrutiert wurden. In Ländern, in denen es möglich ist, Geburtskohorten zu identifizieren, führt dies sehr schnell zu einer nach Alter, Geschlecht und anderen Merkmalen geschichteten Kohorte. In Deutschland, in dem dies nur über die lokalen Meldeämter möglich ist [7] und unter den erschwerten Bedingungen einer unklaren und (noch) nicht abschätzbaren Bereitschaft, an biomedizinischen Untersuchungen teilzunehmen, dürfte es gerade zu Beginn mehr dem Zufall überlassen sein, welche soziografischen Merkmale die Kohorte aufweisen wird. Rekrutierungswege sind hier Zeitungsanzeigen und soziale Medien (z. B. Zwillingsgruppen bei Facebook).

## Die Tübinger *TwinHealth*-Initiative

**Figure 1:**
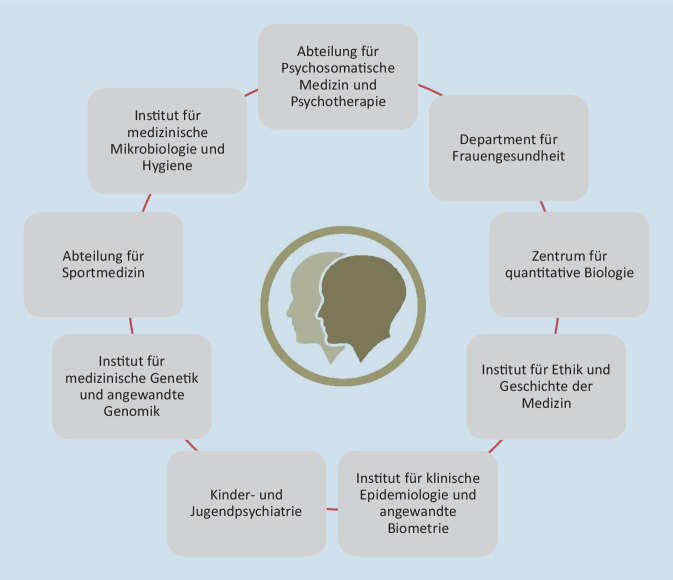

Die Tübinger *TwinHealth*-Initiative begann 2016 mit einer sehr eingeschränkten Forschungsfrage von 2 Gründungsmitgliedern anlässlich der Planung einer Studie zur Placeboanalgesie [20] und der klassischen Frage nach „Anlage oder Umwelt“. Die technischen Schwierigkeiten bei der Rekrutierung von Zwillingen über eine vorhandene Kohorte fernab von Tübingen nötigten zu einer Rekrutierung vor Ort. Dies führte im Jahr 2016 zur Überlegung, die Zwillinge nicht nur für diese, sondern auch für andere Studien zu rekrutieren, und im weiteren Verlauf zur Bündelung von Projekten an Kliniken und Instituten mit Interesse an einer koordinierten Zwillingsforschung in Form des *TwinHealth*-Konsortiums (Abb. 1).

Tübingen wird beim Aufbau des Zwillingskollektivs alle 3 oben skizzierten Wege gehen – dies soll im Folgenden an den derzeit laufenden Studien in diesen 3 Bereichen exemplifiziert werden:

### Neugeborene Zwillinge

An der Tübinger Frauenklinik kommen zurzeit etwa 300 Zwillingspaare pro Jahr zur Welt (Stand 2020). Die Mütter werden bereits während der Schwangerschaft engmaschig betreut. Es besteht ein 2020 bewilligtes EU-Projekt (*PRETWINSCREEN*), an dem die Tübinger Frauenklinik beteiligt ist und das gemäß seinem Titel „Develop a multi-disciplinary approach for a personalized prenatal diagnostics and care for twin pregnancies“ auf der Basis personalisierter pränataler Informationen bei Zwillingsschwangerschaften diagnostische und therapeutische Tools entwickelt. Die über dieses Projekt rekrutierten Zwillinge bzw. ihre Eltern sollen im Verlauf der postnatalen Entwicklung in ein weiteres Projekt (*IMA-TWIN; Intestinal Microbiome Analysis in the Tübingen TWIN Cohort*) eingeschlossen werden, das bereits bewilligt ist. Hier sollen u. a. der Einfluss des frühkindlichen intestinalen Mikrobioms auf die Frequenz und den Schweregrad von kindlichen Koliken, auf die Inzidenz gastrointestinaler Infektionen, auf das Impfansprechen, auf das frühkindliche Schlafverhalten und auf das kindliche Temperament sowie der Einfluss des väterlichen und des mütterlichen intestinalen Mikrobioms auf das kindliche intestinale Mikrobiom untersucht werden.

Zwillinge aus beiden Projekten sind Teil des *TwinHealth*-Kollektivs und können im Verlauf für weitere spezifische Studien kontaktiert werden, sofern Zustimmung vorliegt.

### Gesunde erwachsene Zwillinge

*TwinFIT *ist ein Projekt der Abteilung Sportmedizin und untersucht, inwieweit neben der Veranlagung äußere Einflüsse wie Training Krankheitsrisiken und die körperliche Fitness bestimmen. Außerdem wird untersucht, welchen individuellen Einfluss regelmäßige körperliche Aktivität auf Leistungsfähigkeit und kardiometabole Gesundheit hat. Detailliertere Kenntnisse über mögliche Determinanten einer solchermaßen individuell variablen Trainierbarkeit und körperlichen Fitness könnten dazu beitragen, existierende Empfehlungen zu einer regelmäßigen körperlichen Aktivität auf eine personalisiertere Grundlage zu stellen. Neben Umweltfaktoren wie Aufwachsen in einem bewegungsfördernden oder -abstinenten Umfeld, individuellen Erfahrungen mit körperlicher Aktivität und Sport sowie der Ausprägung der intrinsischen körperlichen Fitness gibt es klare Hinweise, dass genetische Faktoren nicht nur das Ansprechen auf ein körperliches Training beeinflussen, sondern dass die Fitness selbst in Teilen genetisch determiniert ist. *TwinFIT* ist im deutschen Sprachraum das erste große sportmedizinische Zwillingskollektiv. Die Studie läuft bei hoher Rekrutierungsrate über Annoncen erfolgreich seit November 2019, musste jedoch aufgrund der COVID-19-Pandemie zwischenzeitlich immer wieder unterbrochen werden.

### Krankheitskonkordanz und -diskordanz bei Zwillingen

Am Universitätsklinikum Tübingen (UKT) werden jährlich stationär ca. 75.000 und ambulant ca. 366.000 Behandlungsfälle versorgt, monatlich sind dies demzufolge etwa 37.000 Behandlungsfälle. Gemessen an der globalen Inzidenz von Zwillingsgeburten in Deutschland, die zwischen 0,5 % und 1,5 % variiert (je nach Geburtskohorte), müssten unter diesen Patientinnen und Patienten monatlich zwischen 180 und 550 Personen mit einem Mehrlingsstatus sein. Selbst wenn man annimmt, dass sich diese 37.000 Behandlungsfälle in jedem Monat nicht auf 12 × 37.000 Patientinnen und Patienten im Jahr aufaddieren (z. B. wegen mehrfacher Vorstellungen/Aufnahme in verschiedenen Kliniken im Jahresverlauf), ist doch anzunehmen, dass sich eine beträchtliche Anzahl von Zwillingen unter den Behandelten am UKT befindet. Auf der Basis dieser Annahme wurde die Erhebung des Zwillingsstatus ab August 2019 in die Stammdatenerhebung bei ambulanter oder stationärer Aufnahme ins Klinikum eingeschlossen, wenngleich die Beantwortung, ebenso wie die nach Konfession und Familienstatus, auf Freiwilligkeit beruht. Eine aktuelle Abfrage ergab eine Anzahl von bislang 3300 erfassten Personen mit Mehrlingsstatus. Die Ethikkommission sowie der Datenschutzbeauftragte des UKT haben dem Projekt *TwinCORD* zugestimmt, für das diese Patientinnen und Patienten einmalig kontaktiert werden können. Fragen schließen den Anlass der Konsultation, Konkordanz/Diskordanz der vorliegenden Erkrankung beim anderen Zwilling, andere konkordante oder diskordante Erkrankungen in der Vergangenheit und die Bereitschaft, sich für die Zwillingskohorte registrieren zu lassen, ein. Projektstart war August 2020. Das *TwinHealth*-Konsortium ist sich darüber im Klaren, dass hinreichende Daten zunächst nur für Krankheiten mit hoher Prävalenz in der Bevölkerung erhoben werden können. Für seltene Erkrankungen wird dies bedeuten, dass eine ausreichende Fallzahl erst mit der Zeit erreicht wird oder dass andere methodische Vorgehensweisen, wie z. B. der paarweise Vergleich des gesamten Genoms zwischen eineiigen Zwillingen (s. oben), notwendig werden. Das UKT ist unseres Wissens die einzige Klinik in Deutschland, die den Zwillingsstatus ihrer Patientinnen und Patienten routinemäßig erhebt.

*TwinHealth* befindet sich 3 Jahre nach seiner Initiierung in der Realisierungsphase. Fördermittel für den Aufbau der Datenbank und Biobank stehen bereit. Die langfristige Finanzierung von *TwinHealth* muss aus Eigenmitteln der beteiligten Institute und im Rahmen laufender und neuer Einzelprojekte erfolgen. Aktuell werden bereits Zwillinge über alle 3 oben beschriebenen Rekrutierungswege eingeschlossen. Die eigens von der *TwinHealth*-Initiative gestaltete Webseite www.Zwillingsgesundheit.de (www.twinhealth.de) informiert und dient der Rekrutierung. Im Sommersemester 2021 wird es im Rahmen des Studium generale an der Eberhard Karls Universität Tübingen weitere Informationsveranstaltungen für Interessierte geben.

In interdisziplinärer Zusammenarbeit der medizinischen Kliniken, Psychologischen, Biologischen und Biometrischen Fakultäten sowie des Ethik-Instituts wurden erste Schritte unternommen und Projekte begonnen, um langfristig eine Zwillingskohorte für biomedizinische Projekte und Fragestellungen aufzubauen, die weit über den Raum Tübingen und Zeithorizont der beteiligten Wissenschaftlerinnen und Wissenschaftler hinaus reichen soll.
